# Fetomaternal Doppler sonography for the prediction of perinatal outcome in term pregnancies complicated by gestational diabetes mellitus: does it have potential?

**DOI:** 10.1055/a-2554-0806

**Published:** 2025-04-29

**Authors:** Oliver Graupner, Caroline Rath, Linda Lecker, Jochen Ritgen, Bernhard Haller, Christian Enzensberger

**Affiliations:** 1 39058 Department of Obstetrics and Gynecology, University Hospital Aachen, Aachen, Germany; 2 27190 Department of Obstetrics and Gynecology, Klinikum rechts der Isar der Technischen Universität München, Munchen, Germany; 3 Praenatalplus, Cologne, Germany; 4 Institute for Medical Informatics, Statistics and Epidemiology (IMedIS), University Hospital rechts der Isar, Technical University of Munich, Munich, Germany

**Keywords:** fetomaternal Doppler, cerebroplacental ratio, uterine artery Doppler, adverse perinatal outcome, gestational diabetes mellitus

## Abstract

**Purpose:**

Little is known about the benefit and interpretation of fetomaternal Doppler sonography in GDM for the prediction of an adverse perinatal outcome (APO). The aim of this study was to examine the performance of fetomaternal Doppler for APO prediction in pregnancies with GDM at term.

**Materials and Methods:**

This is a retrospective cohort study of singleton, non-anomalous fetuses of women with GDM, who primarily had a vaginal delivery attempt. Study inclusion also required no other major fetomaternal abnormalities that make placental dysfunction likely. Data on fetomaternal Doppler sonography including umbilical artery pulsatility index (PI), middle cerebral artery (MCA) PI, cerebroplacental ratio (CPR), mean uterine artery PI, cerebro-placental-uterine ratio (CPUR) was collected from 37+0 weeks on. Multivariate logistic regression analyses were performed using maternal characteristics, neonatal characteristics, and Doppler ultrasound parameters as independent variables with CAPO as a binary outcome.

**Results:**

A total of n=88 cases were included. Nulliparity (p=0.032) and CPUR (p=0.052) were independent predictors of CAPO. However, CPUR had borderline significance. All other Doppler indices were not independent predictors of CAPO. The ability of CPUR alone (AUC=0.65, 95% CI 0.51 to 0.80) to discriminate between GDM pregnancies with and without CAPO was poor.

**Conclusion:**

This study shows that there is no significant clinical relationship between fetomaternal Doppler indices and CAPO among pregnancies with GDM. This raises the question regarding the extent to which fetomaternal Doppler indices, which reflect placental function, can be helpful for CAPO prediction in GDM pregnancies.

## Introduction

The worldwide prevalence of gestational diabetes mellitus (GDM) has increased significantly in the last 15 years due to changes in GDM screening and the increase in essential risk factors such as maternal age and obesity 1 . Pregnancies complicated by GDM are associated with significant risks of neonatal and maternal adverse outcomes including higher rates of fetal macrosomia, acidosis, neonatal intensive care unit (NICU) admission, and operative delivery (OD) due to intrapartum fetal compromise (IFC) 2 3 . The underlying pathophysiology is multifactorial and only moderately understood to date. Two main pathogenic mechanisms have been proposed so far: First, altered vascularization in the placenta, and second, maternal hyperglycemia increasing fetal oxygen demands 4 . Moreover, women with GDM have evidence of early vascular disease and remodeling 5 , which may contribute not only to their long-term cardiovascular risk but also to a higher risk of an adverse perinatal outcome (APO).

Usually, fetomaternal Doppler sonography is not indicated because of the GDM diagnosis alone, unless there are other obstetric risk factors that require a Doppler sonographic evaluation 1 . However, two retrospective cohort studies with large sample sizes (over n=1,000 GDM pregnancies) showed an increased risk of an APO in the presence of a reduced cerebroplacental ratio (CPR) 6 7 . In a recently published systematic review on the prognostic accuracy of fetoplacental Doppler ultrasound in APO prediction of pregnancies complicated by GDM and preexisting DM, the umbilical artery pulsatility index (UA-PI) had higher sensitivity for APO compared with the CPR and middle cerebral artery (MCA) PI. Nonetheless, there are still significant variations in the time interval between Doppler and birth, GDM severity, definitions of APO, and Doppler thresholds used for test positivity 3 . Furthermore, heterogeneous patient groups including GDM cases with planned cesarean delivery (CD) and/or small-for-gestational-age (SGA) fetuses are major limitations of previous studies. Against the background of a physiological reduction in uteroplacental perfusion (about 60%) during uterine contraction 8 , the mode of delivery appears to be essential in the interpretation of data regarding fetoplacental Doppler changes and APO. Furthermore, it seems necessary to rule out cases with indications of placental dysfunction (PD) such as SGA or hypertensive disorders of pregnancy (HDP) from which we know that changes in fetomaternal Doppler indices occur. To the best of our knowledge, there are not yet any studies on the role of uterine Doppler for APO prediction in GDM pregnancies. Based on current evidence of GDM-mediated maternal vascular changes 5 , however, examination of the whole maternal placental compartment appears important. Therefore, the aim of this study is to investigate the performance of fetomaternal Doppler for APO prediction in GDM pregnancies beyond 37+0 weeks of gestation.

## Materials and Methods

This is a retrospective, single-center cohort study. Some of the study cohort was previously analyzed regarding the role of ductus venosus Doppler sonography for APO prediction in term pregnancies complicated by GDM 9 . Pregnancies with a diagnosis of GDM (on diet: dGDM or requiring insulin: iGDM) based on a 75g glucose oral tolerance test in which fetomaternal Doppler was examined from 37+0 weeks on were included. Data were obtained between 10/2019 and 07/2022. Cases with evidence of chromosomal or morphological fetal anomalies, twin pregnancies, or other fetomaternal conditions with a possible effect on fetomaternal hemodynamics, such as HDP or SGA (birth weight (BW)<10 ^th^ percentile), were excluded from the analysis.

In this study, we focused on hypoxia-related APO, which can possibly be predicted by prenatal fetomaternal Doppler sonography. Therefore, cases with shoulder dystocia (often an unexpected acute event with high hypoxia risk) and neonatal hypoglycemia (frequent hypoglycemia-related and non-hypoxia-related need for transfer to neonatal intensive care unit) were also excluded. Furthermore, only cases with a primary vaginal delivery attempt (exclusion of cases with elective CD) were included.

All GDM pregnancies were monitored and treated (iGDM: induction of labor at the latest 40+0 weeks of gestation, dGDM: induction of labor at the latest 41+0 weeks of gestation) following the recommendations of national guidelines 1 .

The study was approved by the institutional review board/ethics committee of the University Hospital Aachen.

Fetomaternal Doppler examinations were performed using a Voluson E10, E8, or S10 (GE Medical Systems, Solingen, NRW, Germany) with a 2–8 MHz convex probe including the umbilical artery (UA) pulsatility index (PI), middle cerebral artery (MCA) PI, and mean uterine artery (mUtA) PI in all cases. Doppler measurements were performed following the recommendations of the Deutsche Gesellschaft für Ultraschall in der Medizin (DEGUM) 10 . The cerebroplacental ratio (CPR) was calculated as MCA-PI/UA-PI. mUtA-PI was calculated as the average PI of the right and left uterine arteries (UtA). The cerebral-placental-uterine ratio (CPUR), which combines information from the uterine, placental, and fetal vessels as first described in late-onset FGR fetuses 11 , was calculated as CPR/mUtA-PI. mUtA-PI and UA-PI were defined as pathological when they were>95th percentile 12 13 . MCA-PI and CPR were defined as pathological when they were<5th percentile 13 . In case of more than one Doppler examination, the closest examination to delivery was used.

We used IBM SPSS statistics (Version 27.0 for Windows) and R version 4.4.1 for statistical analysis. Analysis of the characteristics of the entire study population according to CAPO was carried out. Quantitative data are shown as means and standard deviations. Categorical data are presented as absolute and relative frequencies. For comparison between groups, the Mann–Whitney *U* test for continuous variables and the Pearson Chi-squared test for categorical variables were used. All statistical tests were two-sided and a p-value<0.05 was considered statistically significant.

Furthermore, multivariate logistic regression analyses were performed using maternal characteristics (body mass index, nulliparous, insulin therapy, induction of labor), neonatal characteristics (gestational age at delivery, birth weight), and ultrasound (UA-PI, MCA-PI, mUtA-PI, CPR, CPUR) parameters as independent variables with CAPO as a binary outcome. CPUR and potential confounders (BMI, nulliparity, insulin therapy, induction of labor, GA at delivery and birth weight) were included in the regression model as independent variables. Finally, the prognostic value of UA-PI, MCA-PI, mUtA-PI, CPR, and CPUR as a continuous variable alone and combined (multivariate logistic regression) to predict CAPO was assessed using the receiver operating characteristic curve (ROC).

Adverse perinatal outcomes of interest were those related to hypoxia. Hence, the presence of at least one of the following APO parameters was defined as composite APO (CAPO):

- Emergency operative delivery (OD) due to intrapartum fetal compromise (IFC)
- Admission to the neonatal intensive care unit (NICU)
- Umbilical cord arterial pH≤7.10
- 5 min. APGAR≤5

The diagnosis of IFC was made based on abnormal fetal heart rate (FHR) patterns and/or pH value≤7.20 of fetal blood gas analysis (scalp). Cardiotocography (CTG)-based fetal heart rate (FHR) patterns were classified according to the International Federation of Gynecology and Obstetrics (FIGO) criteria 14 . OD was defined as CD or instrumental vaginal delivery (IVD).

## Results

This study included n=88 GDM pregnancies (n=56 with dGDM, n=32 with iGDM), which were examined ≥37+0 weeks of gestation. The mean gestational age at the time of examination and at delivery was 38.56+1.06 weeks and 39.69+0.74 weeks, respectively. Overall, CAPO occurred in 15 of 88 cases (17%), whereby there was no case with a 5-min. APGAR value≤5.

The mean CPUR (2.37±0.82 vs. 2.87±0.98, p=0.048) was significantly lower in the CAPO group compared to the non-CAPO. All other Doppler indices (UA-PI, MCA-PI, CPR, mUtA-PI) did not show any significant difference between the CAPO and the non-CAPO group. APO frequencies, baseline characteristics of the study cohort and Doppler results are displayed in Table 1 .

**Table:** **Table 1** APO frequencies and baseline characteristics of n=88 GDM pregnancies at≥37+0 weeks of gestation.

	Non-CAPO (n=73)	CAPO (n=15)	p-value
Maternal age [years] (mean)	34.01±4.56	33.27±6.09	0.588
BMI [kg/m ^2^ ] (mean)	27.15±5.60	29.77±7.69	0.128
Nulliparous (n,%)	24 (32.9%)	10 (66.7%)	0.014*
Previous cesarean section (n,%)	11 (15.1%)	1 (6.7%)	0.388
Insulin therapy (n,%)	24 (32.9%)	8 (53.3%)	0.134
GA at the time of examination [weeks] (mean)	38.55±1.06	38.59±1.06	0.909
UA-PI (mean)	0.80±0.14	0.85±0.15	0.168
MCA-PI (mean)	1.49±0.30	1.41±0.22	0.331
CPR (mean)	1.89±0.39	1.73±0.43	0.141
CPUR (mean)	2.87±0.98	2.37±0.82	0.048*
mUtA-PI (mean)	0.70±0.20	0.79±0.29	0.137
Polyhydramnios (SDP>8cm) (n,%)	7 (9.6%)	2 (13.3%)	0.663
GA at delivery [weeks] (mean)	39.66±0.72	39.81±0.87	0.471
Induction of labor (n,%)	33 (45.2%)	11 (73.3%)	0.047*
Spontaneous vaginal delivery (n,%)	53 (72.6%)	4 (26.7%)	<0.001*
Secondary cesarean section (n,%)	16 (21.9%)	6 (40.0%)	0.141
Birth weight [kg] (mean)	3558±413	3662±325	0.364
LGA (n,%)	14 (19.2%)	4 (26.7%)	0.513
Operative delivery due to IFC (n,%)	0 (0%)	10 (66.7%)	
5-minute APGAR≤5 (n,%)	0 (0%)	0 (0%)	
Umbilical cord artery pH≤7.10 (n,%)	0 (0%)	3 (20%)	
NICU (n,%)	0 (0%)	6 (40%)	

BMI: body mass index, GA: gestational age, PI: pulsatility index, P: percentile, UA: umbilical artery, MCA: middle cerebral artery, mUtA: mean uterine artery, CPR: cerebroplacental ratio, SDP: single deepest pocket, AC: abdominal circumference, IFC: intrapartum fetal compromise, APO: adverse perinatal outcome, CAPO: combined adverse perinatal outcome, LGA: large for gestational age, NICU: neonatal intensive care unit. Data are presented as *n* (%) or mean±SD. A *p* -value<0.05 was considered statistically significant*

**Fig. 1:**
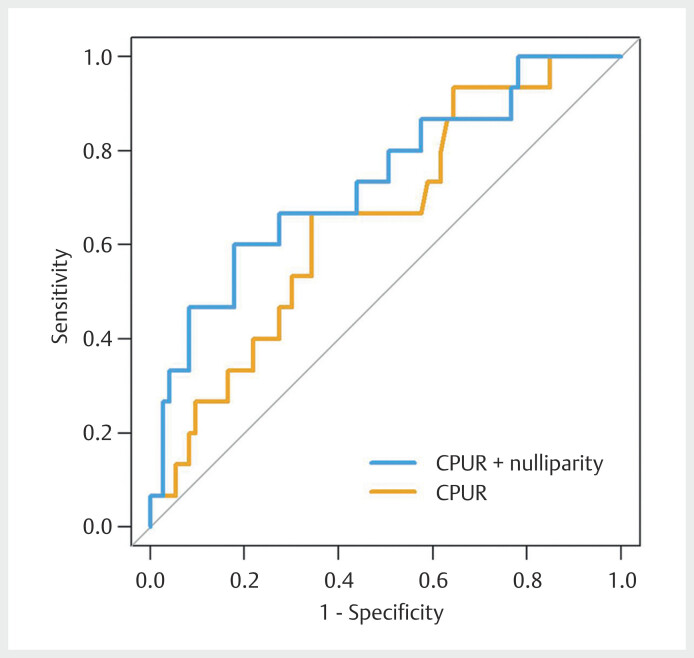
Receiver operating characteristic curve analysis of cerebro-placental-uterine ratio (CPUR) alone (yellow line) and combined (blue line) to predict composite adverse perinatal outcome (CAPO).

Results of univariate logistic regression revealed that only nulliparity is a significant predictor of CAPO (p=0.019) with CPUR (p=0.058) and induction of labor (p=0.056) reaching only borderline significance ( Table 2 ). Multivariate logistic regression showed that only nulliparity (p=0.032) was an independent predictor of CAPO, however with borderline significance for CPUR (p=0.052) ( Table 3 ). All other Doppler indices (UA-PI, MCA-PI, CPR, mUtA-PI) were not independent predictors of CAPO ( Table 3 ). Finally, the ability of CPUR alone (AUC=0.65, 95% CI 0.51 to 0.80) and combined (CPUR and nulliparity: AUC=0.73, 95% CI 0.59 to 0.88) to discriminate between GDM pregnancies with and without CAPO was poor ( Fig. 1 ).

**Table:** **Table 2** Univariate logistic regression of predictors of CAPO.

	Odds ratios	95% CI	*p* -value
BMI (kg/m2)	1.069	0.980–1.167	0.133
Nulliparity	4.083	1.256–3.280	0.019*
Insulin therapy	2.333	0.757–7.193	0.140
Induction of labor	3.333	0.971–11.446	0.056
GA at the time of delivery	1.342	0.607–2.965	0.467
Birth weight	1.001	0.999–1.002	0.362
UA-PI	1.316	0.889–1.949	0.170
MCA-PI	2.736	0.364–20.537	0.328
mUtA-PI	5.491	0.540–55.814	0.150
CPR	3.086	0.679–14.018	0.144
CPUR	1.954	0.977–3.909	0.058

BMI: body mass index, GA: gestational age, PI: pulsatility index, UA: umbilical artery, MCA: middle cerebral artery, mUtA: mean uterine artery, CPR: cerebroplacental ratio, CPUR: cerebro-placental-uterine ratio, CAPO: combined adverse perinatal outcome; A *p* -value<0.05 was considered statistically significant*

**Table:** **Table 3** Multivariate logistic regression of fetomaternal Doppler indices regarding CAPO.

	Unadjusted			Adjusted*		
	Odds ratios	95% CI	*p* -value	Odds ratios	95% CI	*p* -value
UA-PI	1.316	0.889–1.949	0.170	1.529	0.929–2.517	0.095
MCA-PI	2.736	0.364–20.537	0.328	1.685	0.178–15.938	0.649
mUtA-PI	5.491	0.540–55.814	0.150	11.618	0.709–190.420	0.086
CPR	3.086	0.679–14.018	0.144	2.793	0.539–14.461	0.221
CPUR	1.954	0.977–3.909	0.058	2.133	0.994–4.575	0.052

The five Doppler indices were not included in the model together, but individually in combination with the potential confounders. ^*^ Adjusted for: body mass index, nulliparity, insulin therapy, induction of labor, gestational age at birth and birth weight. PI: pulsatility index, UA: umbilical artery, MCA: middle cerebral artery, mUtA: mean uterine artery, CPR: cerebroplacental ratio, CPUR: cerebro-placental-uterine ratio, CAPO: combined adverse perinatal outcome; A *p* -value<0.05 was considered statistically significant*.

## Discussion

The aim of the study was to investigate the performance of fetomaternal Doppler sonography regarding APO prediction in a clearly selected GDM cohort at term (≥37+0 weeks of gestation). Regarding fetomaternal Doppler indices (UA-PI, MCA-PI, CPR, mUtA-PI, CPUR), we observed that none of the Doppler indices clearly helped to differentiate between CAPO and non-CAPO cases. Instead, we found that only nulliparity was significantly predictive of CAPO. To date, the implications of an abnormal UA, MCA, and mUtA Doppler measurement in the daily routine are mainly used in (late-onset) FGR as an indication for the optimal time of delivery 15 . However, the implications of these Doppler indices for GDM pregnancies and their fetuses, who are AGA or often LGA, are less known. In the following, we, therefore, discuss every single Doppler index according to our results and the existing literature.

### Umbilical artery

Generally, UA-PI partly reflects placental vascular resistance. An increased impedance in the UA correlates with the grade of obliteration of the placental vascular bed 16 , which is typical for the pathology of FGR. Placental lesions are associated with an altered vascular prostanoid synthesis in DM, but a rise of UA blood flow resistance is not observed until structural signs of ischemia develop 17 . A large retrospective cohort study on the role of CPR in diabetic pregnancies (preexisting DM and GDM) revealed that CPR is influenced more by the UA-PI rather than the MCA-PI, suggesting that pathogenesis of DM may influence the UA Doppler measurement more than the MCA and therefore may be more linked to APO 18 . Furthermore, in pregnancies complicated by GDM and preexisting DM, the prognostic accuracy of the UA-PI outperformed that of the CPR and MCA-PI 3 . However, it must be considered that women with preexisting DM are more likely to present with end-organ microvascular disease including the uteroplacental circulation 19 . In addition, Gibbons et al. did not exclude cases with hypertension or postnatal SGA, which increases the likelihood of UA-PI interference 6 18 . We excluded cases with postnatally confirmed SGA neonates as well as HDP cases. In our study, UA-PI was not an independent predictor for CAPO, which is in accordance with previous findings 20 .

### Middle cerebral artery

Recently published systematic meta-analyses confirmed an association between low cerebral impedance (reflected by lower CPR or MCA-PI) and APO in low and high-risk pregnancies. However, overall the prediction rate was only moderate 21 22 . MCA Doppler shows so-called “brain sparing” as a fetal adaptation to intrauterine hypoxia, which means an increase in the blood supply to the brain (and other vital organs) resulting in a lower MCA-PI. However, data on MCA-PI in fetuses of GDM mothers compared to non-GDM mothers are heterogeneous. While some authors even report an increased MCA-PI in the GDM cohort, a recent systematic review concludes that there is no significant difference in MCA-PI (in the cohort of preexisting DM and GDM) compared to healthy controls 19 23 24 . Familiari et al. reported MCA-PI as the best predictor for CAPO in their prospective study including n=130 GDM cases 20 . In our study, MCA-PI was not an independent predictor for CAPO.

### Cerebroplacental ratio

Low CPR, which is calculated as the ratio of the MCA-PI to UA-PI, reflects PD. The CPR is a crucial parameter when it comes to the monitoring and timing of birth in late FGR 15 . In the already mentioned review on the role of fetoplacental Doppler in DM pregnancies, the CPR was the most reported Doppler parameter, with the largest sample sizes. However, the prognostic accuracy of the CPR was worse than that of UA and MCA Doppler measurements across all perinatal outcomes 3 . Two large retrospective cohort studies reported a significant association between a reduced CPR and APO in GDM pregnancies 6 7 . Gibbons et al. measured the CPR between 34+0 and 36+6 gestational weeks in a heterogeneous study group, which means cases with suspected PD such as SGA or HDP were included as well as pregnancies delivered by elective CD 6 . To eliminate a major factor possibly influencing the CPR, Garbagnati et al. excluded SGA fetuses from their analysis. However, cases with HDP and/or elective CD were not excluded. They measured the CPR between 35+0 and 36+6 gestational weeks 7 . In our study, the CPR was not an independent predictor for CAPO.

### Uterine arteries

mUtA-PI is an indirect indicator of placental function depending first on spiral artery invasion in the first and early second trimesters and second on the maternal perfusion of the utero-placental compartment 24 . Intrapartum uterine contraction leads to intermittent compression of the placental vascular bed, thus reducing the uteroplacental perfusion and, therefore, oxygen supply to the fetus 8 . Consequently, antenatal placental function reflected by fetal growth and fetomaternal blood flow is essential when it comes to an adequate fetal response to this physiological stress situation during labor. Until now, mUtA-PI measurement in the third trimester was reported for APO prediction and management of SGA fetuses and/or HDP 15 , both of which represent classic pathologies for PD. However, recent data reveals that an abnormal mUtA-PI in early labor is associated with an increased risk of OD for IFC and APO in low-risk term pregnancies, suggesting that there is evidence of subclinical PD even in AGA fetuses 24 . To date, there are no studies on the role of uterine Doppler for APO prediction in a clearly selected GDM population. Against the background of detectable vascular changes in terms of general remodeling 5 , however, Uta Doppler examination and its use for APO prediction seem important. Recent data on midgestation cardiovascular phenotyping in women who develop GDM and/or HDP revealed no evidence of altered placental perfusion or function reflected by mUtA-PI and serum placental growth factor (PlGF) in the GDM group without HDP 25 . In our study, mUtA-PI was not an independent predictor for CAPO. However, we deliberately excluded GDM cases with HDP because of its expected influence on the mUtA-PI. In fact, it has been recently reported that a most distinct pattern of uterine artery resistance could be observed in GDM cases with HDP compared to those without HDP 26 .

### Cerebro-placental-uterine ratio

To date, the CPUR is a poorly understood Doppler parameter that combines information from the uterine, placental, and fetal vessels. Therefore, it has the potential of an improvement in the detection of subclinical PD (especially compared to CPR). The idea of integrating the maternal side of the placenta into the feto-placental Doppler-based APO risk evaluation led to first studies, which assessed the role of the CPUR in high and low-risk pregnancies 11 25 . However, no study on the role of the CPUR in a GDM population has been published to date. Dall’Asta et al., who examined the CPUR in early labor in low-risk term pregnancies reported a six-fold increase in the rate of OD for IFC as well as a higher rate of APO in cases with a low CPUR, even though the predictive power of the CPUR was low 27 . In our study, the mean CPUR was significantly lower in the CAPO group compared to the non-CAPO group. However, multivariate logistic regression showed only borderline significance for the CPUR as an independent predictor of CAPO with odds ratios only around 2.

There are several limitations of our study. First, this is a retrospective study with a small sample size. Due to the retrospective design, we were not able to assess the role of pregnancy glycemic control. Furthermore, we only focused on APO criteria as surrogate markers for fetal hypoxia. The strength of this study is that we realized an overall assessment of fetomaternal Doppler in an isolated GDM cohort without further pathologies (especially exclusion of confirmed SGA as well as HDP cases). Furthermore, we only included cases where vaginal delivery was attempted since labor activity can make a crucial difference when it comes to possible intrapartum hypoxia processes associated with fetomaternal Doppler changes.

This study shows that there is no significant clinical relationship between fetomaternal Doppler indices and CAPO among pregnancies with GDM. This raises the question regarding the extent to which fetomaternal Doppler indices, which reflect placental function, can be helpful for CAPO prediction in GDM pregnancies.
